# Screening for Poor Self-Reported Sleep Quality at 12 Weeks in Post-Mild Traumatic Brain Injury Patients Using the HF–Age–Gender (HAG) Index

**DOI:** 10.3390/brainsci11111369

**Published:** 2021-10-20

**Authors:** Hon-Ping Ma, Ju-Chi Ou, Kai-Yun Chen, Kuo-Hsing Liao, Shuo-Jhen Kang, Jia-Yi Wang, Yung-Hsiao Chiang, John Chung-Che Wu

**Affiliations:** 1 Department of Emergency Medicine, Shuang Ho Hospital, Taipei Medical University, New Taipei City 235, Taiwan; acls2000@tmu.edu.tw; 2 Department of Emergency Medicine, School of Medicine, Taipei Medical University, Taipei 110, Taiwan; 3 Graduate Institute of Injury Prevention and Control, Taipei Medical University, Taipei 110, Taiwan; 4 Neuroscience Research Center, Taipei Medical University, Taipei 110, Taiwan; juchi.tmu@gmail.com (J.-C.O.); kychen08@tmu.edu.tw (K.-Y.C.); khliao@tmu.edu.tw (K.-H.L.); terbiun@gmail.com (S.-J.K.); jywang2010@tmu.edu.tw (J.-Y.W.); ychiang@tmu.edu.tw (Y.-H.C.); 5 Division of Neurosurgery, Department of Surgery, School of Medicine, College of Medicine, Taipei Medical University, Taipei 110, Taiwan; 6 Graduate Institute of Neural Regenerative Medicine, College of Medical Science and Technology, Taipei Medical University, Taipei 110, Taiwan; 7 Department of Neurosurgery, Wan Fang Hospital, Taipei Medical University, Taipei 116, Taiwan; 8 Division of Neurosurgery, Department of Neurosurgery, Taipei Medical University Hospital, Taipei 110, Taiwan

**Keywords:** mild traumatic brain injury, sleep quality, heart rate variability, Pittsburgh sleep quality index

## Abstract

To identify a screening tool for poor self-reported sleep quality at 12 weeks according to non-invasive measurements and patients’ characteristics in the first week after mild traumatic brain injury (mTBI), data from 473 mTBI participants were collected and follow-ups were performed at 12 weeks. Patients with previous poor self-reported sleep quality prior to the injury were excluded. Patients were then divided into two groups at 12 weeks according to the Pittsburgh Sleep Quality Index based on whether or not they experienced poor sleep quality. The analysis was performed on personal profiles and heart rate variability (HRV) for 1 week. After analyzing the non-invasive measurements and characteristics of mTBI patients who did not complain of poor sleep quality, several factors were found to be relevant to the delayed onset of poor sleep quality, including age, gender, and HRV measurements. The HRV–age–gender (HAG) index was proposed and found to have 100% sensitivity (cut-off, 7; specificity, 0.537) to predicting whether the patient will experience poor sleep quality after mTBI at the 12-week follow-up. The HAG index helps us to identify patients with mTBI who have no sleep quality complaints but are prone to developing poor self-reported sleep quality. Additional interventions to improve sleep quality would be important for these particular patients in the future.

## 1. Introduction

Traumatic brain injury (TBI), often described as a “hidden epidemic”, is a public health problem and a significant cause of death and functional impairment. It is estimated that more than 10 million people are admitted to hospitals annually after TBI [1,2,3]. TBI has three general categories: mild, moderate, and severe. Approximately 80–90% of TBI cases are mild TBI (mTBI) with a Glasgow Coma Scale (GCS) score of 13–15, a loss of consciousness for <30 min, and post-traumatic amnesia for <24 h [4,5]. Most patients with mTBI recover rapidly without any treatment; however, some patients experience persistent cognitive, physical, and emotional impairments, including dizziness, depression, anxiety, and sleep disturbances [4,6,7]. These symptoms negatively impact the long-term outcomes of mTBI patients and subsequently increase the social and economic burden. 

Sleep disturbance is another critical global issue associated with negative impacts on health, work, and quality of life. Patients with sleep disturbance have a higher risk of inflammatory diseases, diabetes mellitus, cardiovascular diseases, and cancers [8,9]. In addition, persistent sleep disturbance induces a deterioration in cognitive, functional, and emotional outcomes following TBI. Kempf et al. conducted a prospective study on 51 TBI patients and found that 67% of those patients had persistent sleep disturbances at 3 years after the TBI [10]. In addition, our previous study showed that 70% of mTBI patients experience an increased risk of sleep problems compared with non-mTBI controls [11]. Moreover, mTBI patients were found to have different sleep durations and subjective sleep quality in self-report questionnaires [11]. Finally, we noted that sleep problems in mTBI patients were worse at later follow-ups [12]. Thus, these studies provide evidence of long-lasting sleep problems in mTBI patients and highlight the emerging need to identify an ideal biomarker for sleep disturbance after mTBI.

Heart rate variability (HRV), which measures the change in heartbeats in a time interval, is an indicator of disease and mortality risk [13] and may be helpful in assessing post-TBI insomnia. HRV analysis methods assess the time and frequency domains. The time-domain measurement includes the mean normal-to-normal (NN) interval, the standard deviation of the NN (SDNN) interval, the square root of the mean squared difference in successive NN intervals, and the percentage of successive differences > 50 ms in intervals between normal heartbeats. The frequency domain typically includes three measures: very low frequency (≤0.04 Hz), low frequency (LF, 0.04–0.15 Hz), and high frequency (HF, 0.15–0.4 Hz). The HF component measures vagal activity, while the LF component is related to a combination of both vagal and sympathetic activities, and the ratio of LF to HF (LF/HF) reflects the cardiac sympathovagal balance [14]. HRV is controlled by the autonomic nervous system, including the sympathetic nervous system (SNS) and the parasympathetic nervous system (PNS). In general, SNS activity increases heart rate, while PNS activity decreases heart rate. Thus, HRV is the final result of the rhythmic, integrated activity of autonomic neurons and measures nervous system competence [15].

There is increasing evidence showing that HRV could be an objective biomarker for various diseases. For example, reduced HRV can predict hypertension, the development of a diabetic neuropathy, cerebrovascular disease, congestive heart failure, and lethal arrhythmic complications after acute myocardial infarction (MI) [16,17]. Patients who had a SDNN < 50 ms and a HRV triangular index score < 15 were at high risk of MI. In addition, Urbanik et al. demonstrated that HRV time parameters are significantly lower in patients with moderate and severe apnea than in those with mild apnea [18]. Recently, a meta-analysis of HRV in patients with major depression (MD) showed that all HRV measures were lower in MD patients than in healthy controls, indicating that lower HRV is a potential cardiovascular risk factor in patients with MD [19]. Our previous study revealed that reduced power of HRV parameters in the frequency domain was found in mTBI patients and was significantly correlated with anxiety and depression [20]; however, the association between HRV and sleep quality remains unclear. Therefore, in this study, we assessed whether HRV might predict post-TBI sleep quality in patients without prior sleep disturbances.

## 2. Methods

### 2.1. Participants

**Figure 1:**
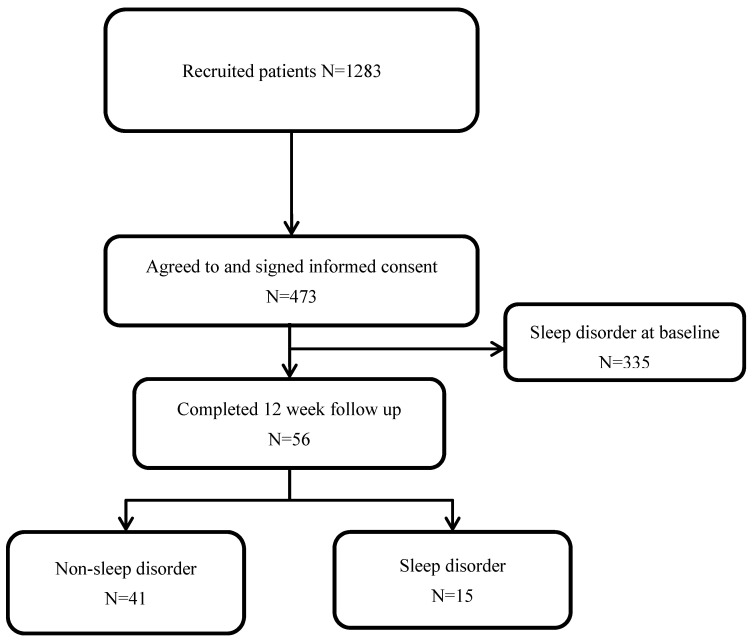
Flow chart of patient inclusion. Inclusion criteria: (1) head injury with GCS > 12; (2) negative brain computed tomography findings; (3) older than 20 years; and (3) no history of brain injury. Exclusion criteria: (1) the patient was pregnant; and (2) the patient suffered from sleep problems at the initial assessment.

We conducted a prospective study on patients with mTBI with a 12-week follow-up. This study was approved by the Taipei Medical University’s Joint Institutional Review Board for the Protection of Human Subjects. Patients were recruited from the emergency departments of three medical centers, and 473 participants agreed to participate in this study and provided informed consent (Figure 1). The severity of TBI is categorized by the GCS, which comprises three parameters: verbal response, eye response, and motor response. A summed score of 3–8 represents severe TBI, and a score of 13 or higher represents mTBI. Patients were recruited according to the following inclusion criteria: (1) head injury with GCS > 12; (2) negative brain computed tomography findings; (3) older than 20 years; and (3) no history of brain injury. Exclusion criteria included the patient (1) being pregnant and (2) suffering from sleep problems at the initial assessment. A flowchart of the study is shown in Figure 1. Among the participants, 138 (29.17%) self-reported non-sleep disorders at the baseline assessment and 56 (40.58%) completed a 12-week assessment. There were no differences in the baseline characteristics between patients who completed and patients who did not complete the 12-week follow-up assessment (Supplementary data Table S1).

### 2.2. Measurement

Pittsburgh Sleep Quality Index (PSQI). The PSQI is a self-report questionnaire that measures an individual’s sleep quality [21]. The Chinese version was used for our patients [22]. The questions assessed 7 aspects of sleep quality, including sleep disturbance, use of sleeping medication, subjective sleep quality, habitual sleep efficiency, sleep latency, sleep duration, and daytime dysfunction. Each aspect scores from 0 (no problem) to 3 (severe), and the total score ranges from 0 to 21 for the PSQI. Thus, a higher score represents a worse sleep quality. The PSQI questionnaires were collected twice (at baseline and at the 12-week follow-up assessment).

Heart rate variability (HRV). HRV was recorded in patients during a short-term resting phase of 5 min via a HRV monitor (LR8Z11). Heart rate (HR), the time domain of HRV, and the frequency domain of HRV were measured. The spectrum was divided into three different frequency bands: 0.0033–0.04 Hz (the very low frequency (VLF) band), 0.04–0.15 Hz (the low frequency (LF) band), and 0.15–0.4 Hz (the high frequency (HF) band). A wider spectrum ranges from 0.0033 Hz to 1.0 Hz (total power (TP)). In addition, the ratios of LF to HF (LF/HF) were calculated and analyzed in our study. The HRV data were collected at the baseline assessment.

### 2.3. Statistical Analysis

Comparison of the patients’ characteristics between groups used a non-parametric method, the Mann–Whitney U test for continuous data, and the chi-square test for categorical data. The new scoring profile, the HF–age–gender (HAG) index, was proposed via a multistep process. First, the corresponding binary factors were generated according to the optimal cut-off point from their corresponding receiver operator characteristic (ROC) curves. The selection of three significantly different factors allowed for the formulation of the HAG index. Next, the weights of the predictors were calculated via logistic regression and then rounded up to the nearest integer. Finally, the sensitivity, specificity, negative predictive value (NPV), positive predictive value (PPV), ROC curve, and optimal cut-off point were evaluated for each factor. The analyses were performed using R software and *p*-values less than 0.05 were considered statistically significant.

## 3. Results

### 3.1. Patients’ Characteristics

Baseline patient characteristics are shown in Table 1. Forty-one (73.21%) mTBI patients reported no sleep disorder at 12 weeks, while 15 (26.79%) mTBI patients reported a sleep disorder. The patients’ education, GCS score, and injury mechanism were not significantly different between the sleep-disorder and non-sleep-disorder groups. The average ages were 38.51 and 51.67 years for non-sleep-disorder and sleep-disorder patients, respectively. Approximately 41% and 87% of the patients were female in the non-sleep-disorder and sleep-disorder groups, respectively.

### 3.2. HRV Parameters

The comparison of HRV parameters between the non-sleep-disorder and sleep-disorder groups is shown in Table 2. There were no differences in all HRV parameters, except for HF. The average value of HF in the non-sleep-disorder group was higher than that in the sleep-disorder group.

### 3.3. ROC Curve of HRV Parameters

**Figure 2:**
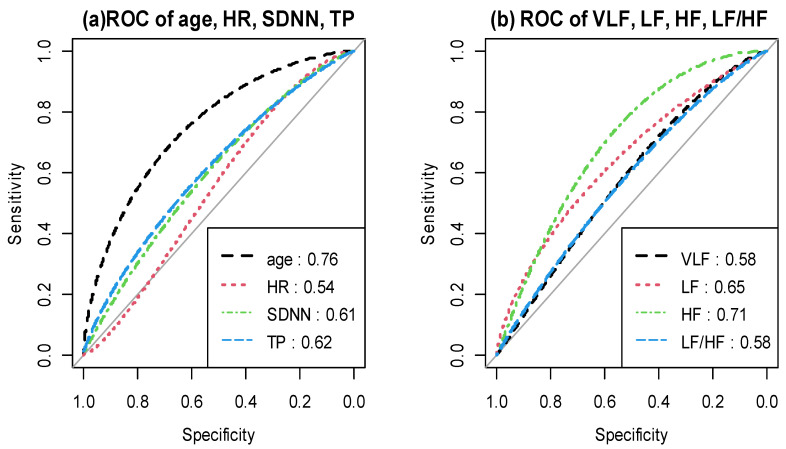
(**a**) ROC curve for HRV parameters (Age: dashed line with an AUC of 0.76; Heart rate (HR): dotted line with an AUC of 0.54; Standard deviation of the NN interval (SDNN): dot-dashed line with an AUC of 0.61; Total power (TP): long-dashed line with an AUC of 0.62. (**b**) ROC curve for HRV parameters (very low frequency (VLF): dashed line with an AUC of 0.58; low frequency (LF): dotted line with an AUC of 0.65; high frequency (HF): dot-dashed line with an AUC of 0.71; LF/HF: long-dashed line with an AUC of 0.58).

The evaluation of HRV parameters’ prediction performance using the ROC curve is shown in Figure 2, and the performance values are listed in Supplementary data Table S2. Among the HRV parameters, the AUC of HF was the highest (0.71) with a sensitivity of 0.73, and the AUC of HR was the lowest.

### 3.4. HF–Age–Gender (HAG) Index

The HAG index was proposed to evaluate the risk of sleep disorders in patients with mTBI according to the HRV performance after an injury. Three significant predictors, age, gender, and HF, were selected for the HAG index, and the results are shown in Table 3. Based on the results from the ROC curve, the cut-off point of age was set to 50 and that of HF was set at 138. The corresponding odds ratios were 1.90, 9.3, and 4.81 for age, gender, and HF, respectively. The risk scores of these three variables were rounded up to two, nine, and five, respectively.

**Figure 3:**
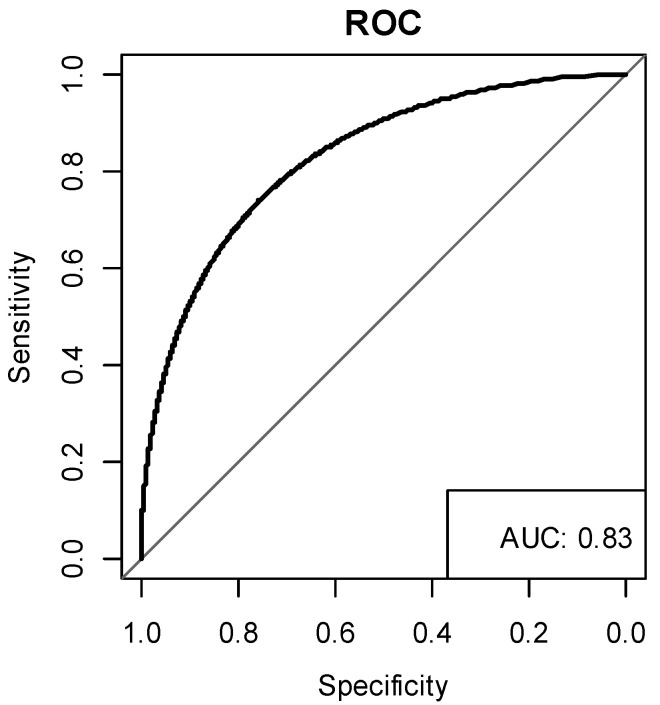
ROC curve of the HAG index with an AUC of 0.83.

The performance of the HAG index was evaluated using the ROC curve (Figure 3). The AUC of the HAG index was 0.83. The corresponding sensitivities and specificities are shown in Table 4. The sensitivity and specificity were 1 and 0.537 when the cut-off point was at 7, which means that a mTBI patient has a high risk of poor sleep quality after 12 weeks post-injury if the HAG index is greater than or equal to 7. According to the cut-off points list from Table 4 and the risk score of factors from Table 3, a female was at high risk of poor sleep quality after 12 weeks post-injury, and a male older than 50 years was at high risk of poor sleep quality after 12 weeks post-injury. In addition, a patient with an HF of less than 138 was more likely to have poor sleep quality after 12 weeks post-injury. Here, we provide the varying cut-off points with the corresponding sensitivity and specificity for the border choice option.

## 4. Discussion

HRV could help to diagnose sleep quality and has been successfully screened for possible referral to sleep specialists. However, post-TBI poor sleep quality occurs many months after TBI, and, currently, no methods are available for predicting these cases of poor sleep quality. Therefore, additional methods are needed to predict the diagnosis of post-TBI poor sleep quality. This report proposed the HAG index to predict poor sleep quality in post-TBI patients without prior poor sleep quality.

Education level is not a significant factor in distinguishing self-reported sleep quality among patients. While it was a borderline influencing factor of poor self-reported sleep quality at the 12-week follow-up, and those in the good self-reported sleep quality group had a longer duration of education than those in the poor self-reported sleep quality group, the statistics in this study only showed a borderline significance (*p* = 0.09) in describing the poor self-reported sleep quality in the TBI patients. Thus, while the findings are compatible with those of Wu et al. that marriage and a higher education level were associated with a decreased risk of poor sleep quality for females [23], its use in our index was avoided due to the borderline significance for the prediction of poor sleep quality in post-TBI patients.

HRV predicts the prognosis of acute and chronic cardiovascular events, and lower HRV is associated with higher cardiovascular mortality [24]. HRV can also be used to detect autonomic nervous system dysfunction, and severe autonomic nervous system dysfunction may shorten the patient’s time to death in terminally ill patients. In addition, higher HRV might predict more prolonged survival in cancer patients due to the effect of vagus nerve activity on cancer progression. Evidence also showed that the interaction of HRV and inflammatory mediators such as TNF-α and IL-6 is associated with cardiovascular mortality in a population-based CARLA cohort study [25]. Moreover, the release of inflammatory cytokines and chemokines following TBI can alter sleep [26,27,28,29,30,31]. Higher levels of peripheral IL-1β in the first 24 h post-injury are related to more significant cumulative sleep in TBI mice [32]. In addition, injections of IL-1β enhance NREM sleep [33] and EEG delta wave activity [34]. However, sleep disruption following TBI may further worsen inflammation and alter the stress-immune pathways [35]. Whether acute or chronic inflammation after TBI is involved in the predictive value of HRV in sleep disturbance requires further examination.

In addition to the various variables in HRV, the HAG index helps to provide prediction values for assessing post-TBI sleep disorders. The HF increased in the group without a sleep disorder, and these values decreased in the group with a sleep disorder. Furthermore, HR did not change in these two groups. While the ratio between the values of TP, LF, HF, VLF, and HR differentiate the group with a sleep disorder and the group without a sleep disorder, the HAG index would be more sensitive in facilitating decision-making in screening for patients with sleep disorders. Finally, in addition to education level and mechanisms of injury, the HAG index shows potential in screening for the presence of sleep disorders in TBI patients without a prior sleep disorder.

Patients with prior sleep disorders were excluded from our study because of the clinical relevance of the need to identify those who would suffer from sleep disorders. Those with sleep disorders prior to TBI are frequently already taking medications or under treatment, and the need for additional treatment or medication is not solely dependent on the method of identification of their sleep disorder. In contrast, only those without prior sleep disorders need early identification of possible subsequent disorders. The HAG index would help to screen for early identification/prediction of individuals who may need follow-up of their TBI condition.

Formulation of the HAG index was initiated after the collection of data in this trial. While the delay may cause a bias in the ultimate result of the index, leading to an imperfect interpretation of the data collected, the HAG index is able to provide a value for the correlation of sleep and HRV results. Clinicians of TBI patients without sleep disorders can utilize this value to understand the sleep quality of patients 12 weeks after the incident. This understanding should also allow clinicians to gain more insight when deciding the post-TBI follow-up duration for patients.

## 5. Limitations

HRV can be used to screen for people with poor sleep quality and may be helpful for screening post-TBI sleep disorders. However, in this study, mTBI patients who reported poor sleep quality at the baseline assessment were excluded, and the result cannot be generalized to all mTBI patients. Further examination with trials involving a larger number of patients and additional research including post-TBI patients with prior sleep problems should reveal additional information regarding the utility of the HAG index in interpreting data on the poor sleep quality of the patient.

## 6. Conclusions

In summary, this study established the HAG index to evaluate the risk of poor sleep quality after mTBI. HAG ≥ 7 indicates that an mTBI patient has a higher risk of poor sleep quality 12 weeks post-injury. Thus, this index is beneficial for screening patients who may suffer from poor sleep quality after TBI. Additionally, it may be an indicator for monitoring sleep improvement during rehabilitation.

## Figures and Tables

**Table 1:** Baseline characteristics between sleep-disorder and non-sleep-disorder mTBI patients (mean ± standard deviation).

Variables	Non-Sleep Disorder	Sleep Disorder	*p*-Value
Sample size	41	15	
Age (year)	38.51 ± 13.32	51.67 ± 12.11	<0.01 *
Female (N, %)	17 (41.46%)	13 (86.67%)	<0.01 *
Education (year)	14.78 ± 2.63	13.46 ± 3.04	0.09
GCS	40 (97.56%)	13 (86.67%)	0.07
Injury mechanism (N, %)			0.70
Falls	12 (29.27%)	4 (26.67%)	
Traffic accident	17 (41.46%)	8 (53.33%)	
Others	12 (29.27%)	3 (20.00%)	
PSQI at baseline	3.54 ± 1.21	3.87 ± 1.19	0.34
PSQI at 12 weeks	2.90 ± 1.26	8.73 ± 2.46	<0.01 *

*: *p*-value < 0.05; GCS, Glasgow Coma Scale; PSQI, Pittsburgh Sleep Quality Index.

**Table 2:** HRV parameters.

Index	Non-Sleep Disorder	Sleep Disorder	*p*-Value
HR	74.00 ± 10.46	75.73 ± 8.09	0.52
SDNN	41.59 ± 21.39	32.67 ± 13.82	0.21
TP	2224.32 ± 2347.32	1266.07 ± 855.87	0.17
VLF	1054.49 ± 1133.04	711.93 ± 530.81	0.38
LF	718.41 ± 914.37	341.33 ± 290.31	0.09
HF	368.17 ± 388.16	150.87 ± 152.73	0.02 *
LF/HF	2.62 ± 2.02	2.65 ± 1.12	0.36

*: *p*-value < 0.05; HR, heart rate; SDNN, standard deviation of NN intervals; TP, total power; VLF, very low frequency; LF, low frequency; HF, high frequency; LF/HF, ratio of low frequency to high frequency.

**Table 3:** Results of the multivariate logistic regression for the risk profile.

Predictor	Criteria	Estimate	OR	Risk Score
Age	>50	0.64	1.90	2
Gender	Female	2.23	9.30	9
HF	<138	1.57	4.81	5

OR, odds ratio.

**Table 4:** ROC curve result for the HAG index.

Cut-Off Point	Sen	Spe	NPV	PPV
0	1	0	NA	0.268
2	1	0.317	1	0.349
5	1	0.390	1	0.375
7	1	0.537	1	0.441
9	0.867	0.585	0.923	0.433
11	0.600	0.829	0.850	0.562
14	0.600	0.878	0.857	0.643
16	0.533	0.902	0.841	0.667

Sen, sensitivity; Spe, specificity; NPV, negative predictive value; PPV, positive predictive value.

## Data Availability

The data used to support the findings of this study are available from the corresponding author upon request.

## Supplementary Materials

The following are available online at https://www.mdpi.com/article/10.3390/brainsci11111369/s1, Table S1. Dropped out vs. followed up group (mean ± standard deviation); Table S2. ROC result for HRV parameters.

## Clinical Trial Registration Number

ClinicalTrials.gov Identifier: NCT01266356.
