# Supplementary material for: Screening for Poor Self-Reported Sleep Quality at 12 Weeks in Post-Mild Traumatic Brain Injury Patients Using the HF–Age–Gender (HAG) Index
Source: Brain Sci. 2021 Oct 20;11(11):1369. doi: 10.3390/brainsci11111369 (PMC8615360; doi:10.3390/brainsci11111369)
Supplement: Supplementary file 1 [file brainsci-11-01369-s001.zip › brainsci-1393313-SI.pdf]

Supplementary data

Table S1. Dropped out vs followed up group (mean ± standard deviation)

Variables	Followed up	Dropped out	p-value
Sample size	56	82	
Age (year)	42.04±14.17	39.33±15.94	0.18
Female (N, %)	30 (54%)	40 (49%)	0.56
Education (year)	14.46±2.77	14.51±2.58	0.82
GCS =15 (N, %)	53 (95%)	82 (100%)	0.06
Injury mechanism (N, %)			0.45
Falls	16 (28.57%)	29 (35.37%)	
Traffic accident	25 (44.64%)	38 (46.34%)	
Others	15 (26.79%)	15 (18.29%)	
PSQI at baseline	3.62±1.20	3.39±1.33	0.34
HRV parameter			
HR	74.46±9.84	74.61±9.79	0.93
SDNN	39.20±19.93	40.13±18.13	0.67
TP	1967.64±2092.13	1875.54±1767.07	0.86
VLF	952.73±1014.30	906.24±901.99	0.75
LF	617.41±811.11	572.52±591.01	0.83
HF	309.96±353.47	305.12±364.56	0.86
LF/HF	2.63±1.81	2.51±1.87	0.45

GCS: Glasgow Coma Scale; PSQI: Pittsburgh Sleep Quality Index; HR: heart rate; SDNN: standard deviation of NN intervals, TP: total power; VLF: very low frequency; LF: low frequency; HF: high frequency; LF/HF: ratio of low frequency and high frequency

Table S2. ROC result for HRV parameters

Variable	AUC	Cut-off point	Spe	Sen	NPV	PPV
age	0.76	51.5	0.8	0.67	0.87	0.56
HR	0.54	69	0.39	0.87	0.89	0.34
SDNN	0.61	51.5	0.27	1	1	0.33
TP	0.62	2614.50	0.24	1	1	0.33
VLF	0.58	802.50	0.49	0.73	0.83	0.34
LF	0.65	133.50	0.90	0.40	0.80	0.60
HF	0.71	138.00	0.68	0.73	0.88	0.46
LF/HF	0.58	2.7	0.63	0.60	0.81	0.38

AUC: area under ROC curve; Sen: sensitivity; Spe: specificity; NPV: negative predictive value; PPV: positive predictive value; HR: heart rate; SDNN: standard deviation of NN intervals, TP: total power; VLF: very low frequency; LF: low frequency; HF: high frequency; LF/HF: ratio of low frequency and high frequency
